# In silico analysis unveiling potential biomarkers in gallbladder carcinogenesis

**DOI:** 10.1038/s41598-024-61762-4

**Published:** 2024-06-24

**Authors:** Raviranjan Kumar Gupta, Ravi Bhushan, Saket Kumar, Shyam Babu Prasad

**Affiliations:** 1 Department of Zoology, School of Life Sciences, Mahatma Gandhi Central University Bihar (MGCUB), Motihari, 845401 India; 2 Department of Zoology, Munsi Singh College, Motihari, 845401 India; 3 Department of Surgical Gastroenterology, Indira Gandhi Institute of Medical Sciences (IGIMS), Sheikhpura, Patna, India

**Keywords:** Gallbladder cancer (GBC), Biomarkers, Network analysis, TRIP13, NEK2, TPX2, Prognosis, Diagnosis, Treatment, Therapeutic targets, Cancer, Computational biology and bioinformatics, Oncology

## Abstract

Gallbladder cancer (GBC) is a rare but very aggressive most common digestive tract cancer with a high mortality rate due to delayed diagnosis at the advanced stage. Moreover, GBC progression shows asymptomatic characteristics making it impossible to detect at an early stage. In these circumstances, conventional therapy like surgery, chemotherapy, and radiotherapy becomes refractive. However, few studies reported some molecular markers like KRAS (Kirsten Rat Sarcoma) mutation, upregulation of HER2/neu, EGFR (Epidermal Growth Factor Receptor), and microRNAs in GBC. However, the absence of some specific early diagnostic and prognostic markers is the biggest hurdle for the therapy of GBC to date. The present study has been designed to identify some specific molecular markers for precise diagnosis, and prognosis, for successful treatment of the GBC. By In Silico a network-centric analysis of two microarray datasets; (GSE202479) and (GSE13222) from the Gene Expression Omnibus (GEO) database, shows 50 differentially expressed genes (DEGs) associated with GBC. Further network analysis revealed that 12 genes are highly interconnected based on the highest MCODE (Molecular Complex Detection) value, among all three genes; TRIP13 (Thyroid Receptor Interacting Protein), NEK2 (Never in Mitosis gene-A related Kinase 2), and TPX2 (Targeting Protein for Xklp2) having highest network interaction with transcription factors and miRNA suggesting critically associated with GBC. Further survival analysis data corroborate the association of these genes; TRIP13, NEK2, and TPX2 with GBC. Thus, TRIP13, NEK2, and TPX2 genes are significantly correlated with a greater risk of mortality, transforming them from mere biomarkers of the GBC for early detections and may emerge as prognostic markers for treatment.

## Introduction

Gallbladder cancer (GBC) is a rare and highly fatal type malignancy, that represents almost 50% of all digestive tract cancer^1^. It is the fifth most common digestive tract cancer worldwide and has a specific geographic distribution with some ethnic variation^1^. GBC is most prevalent in the northern Indian population in the World living near the rural Gangetic basin, and recording about 1.7% of cancer-related mortality and 220,000 incidence cases in 2018^1,2^. The major etiological factors of GBC include Chronic inflammation and, a history of gallstones (cholelithiasis), and the risk increases with gallstone size, chronicity, and burden of symptoms^2^. GBC is often asymptomatic, detected at the advanced stage when it becomes very aggressive leading to poor prognosis^2,3^. The delay in detection often limits the effectiveness of treatment options. Moreover, the absence of specific molecular markers for early diagnosis and to development of prognostic markers is a major hurdle to managing this disease burden. This has prompted a growing interest in the role of biomarkers as indispensable tools in the battle against gallbladder cancer^4^. The identification of biomarkers, in this context, encompasses a diverse range of molecular, genetic, or biochemical indicators that can be objectively measured in biological samples^5^. Recent advancements in genomic and proteomic technologies could help in the identification of several promising biomarkers associated with gallbladder cancer^6^. By providing valuable insights into the underlying molecular mechanisms and disease progression, they empower clinicians to make informed decisions tailored to each patient's unique profile^7^.

Despite the advancement in transcriptomics and microarray bioinformatics, still there is a very scarce study that shows the potent biomarkers related to GBC. Even in those available studies, most of the studies simply identified DEGs they have not stratified the different stages of GBC and have not identified the early-stage biomarkers. A recent study by Jia et al., 2023 identified KNTC1 and MCM2 as the molecular targets of GBC, however, they have not discussed whether they serve as the early stage biomarkers or not^8^. Another study by Gu et al., 2015 revealed that GBC patients with high TK1 and MMP9 expression levels had worse prognosis^9^. Hence, there is an urgent need to decipher the early-stage biomarkers. The present study aims to identify the most potent early-stage biomarkers in GBC progression.

So, considering the above facts, the present study is designed to find promising novel early diagnostic and prognostic biomarkers of GBC. Further, this study aims to crack the code of GBC by searching for tiny “fingerprints” called molecular markers, which may help in earlier diagnosis, personalized predictions, and serve for more patient-centric treatment plans^10^. In this study, two microarray datasets (GSE202479 and GSE132223) containing samples for early stage, advanced stage, and normal samples were analyzed. A total of 50 DEGs between GBC and non-cancerous tissues were screened from the datasets. Further analysis revealed 12 hub genes and three genes; TRIP13, NEK2, and TPX2 were selected as candidate biomarkers for the diagnosis, treatment, and prognosis of GBC. We have also identified the transcription factor and micro-RNA regulating these biomarkers. These biomarkers may hold the potential to revolutionize the diagnostic landscape and therapeutic approach to aggressive GBC malignancy.

## Material and methods

### Data retrieval

**Figure 1:**
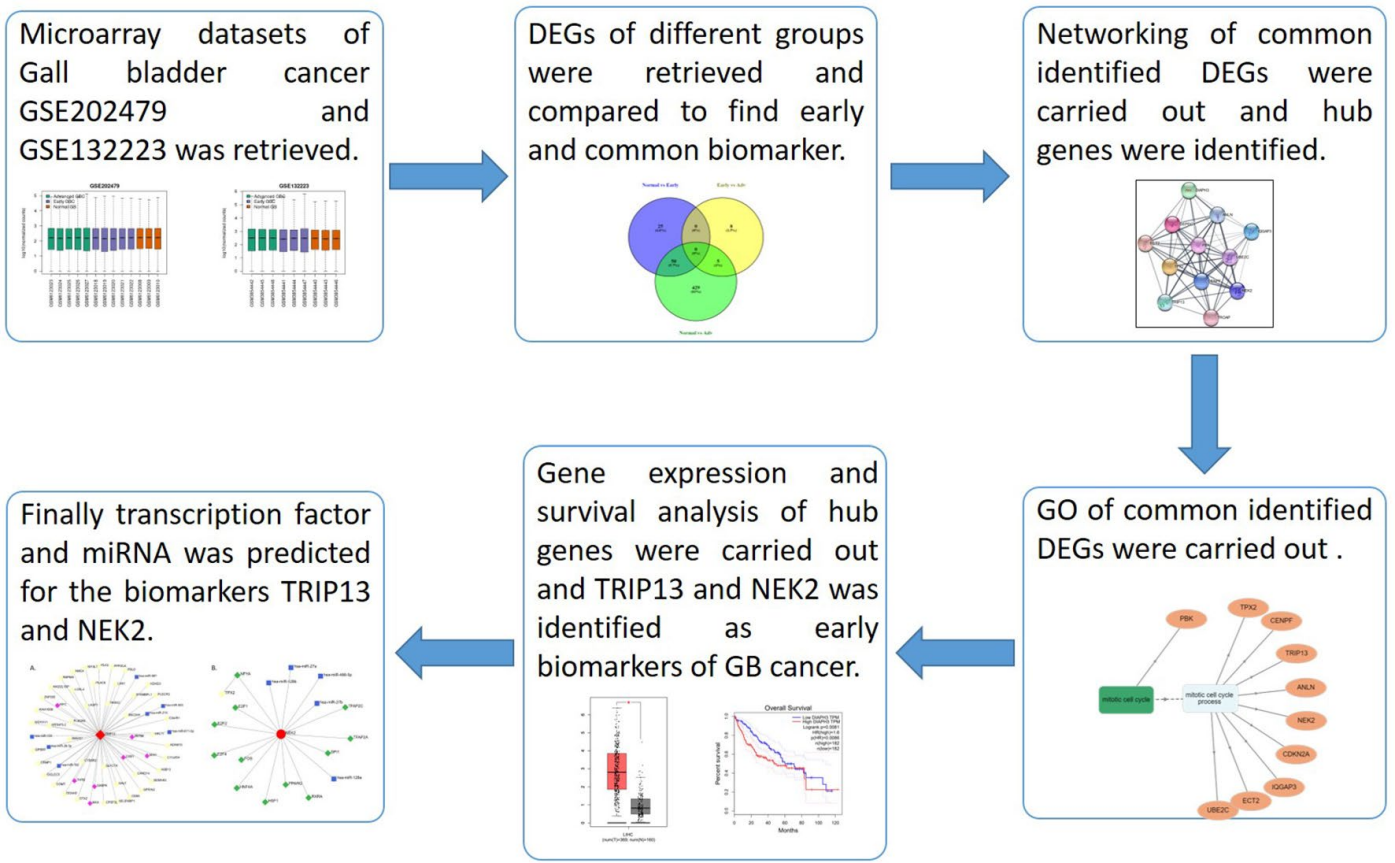
Flow chart showing the overall process adopted in this manuscript to identify the biomarkers.

Two microarray datasets with GSE IDs GSE202479 and GSE132223 were taken from NCBI GEO datasets (https://www.ncbi.nlm.nih.gov/gds). Datasets GSE202479 contain a total of 20 samples, which contain 3 cases of normal gallbladder, 4 cases of gallbladder with chronic inflammation induced by gallstones, 3 cases of gallbladder adenoma, 5 cases of early GBC, 5 cases of advanced GBC. Dataset GSE132223 contains a total of 9 samples containing 3 samples of GBC liver metastatic tumor, 3 samples of primary tumor, and 3 samples of adjacent non-cancerous. The GPL 246,766 platform Illumina NovaSeq 6000 (Homo sapiens) and GPL11154, Illumina NovaSeq 2000 (Homo sapiens) were utilized for GSE202479 and GSE132223 datasets. A series matrix file and other information were also accessed. Whole samples in both datasets were grouped into three groups namely normal, early, and advanced. The whole process of analysis has been outlined and represented in the Fig. 1.

### DEGs identification

**The GEO2R** (https://www.ncbi.nlm.nih.gov/geo/geo2r/) tool was used to divide samples into relevant groups and to compare different groups. The tools GEOquery and limma were used to perform differential expression analysis. GEOquery parses GEO data into R data structures while limma (Linear Models for Microarray Analysis) is a statistical test for identifying differentially expressed genes in microarray data. Logfc_10_ value +/−> 1 and adj *p*-value < 0.05 were used as criteria for sorting of DEG. DEGs among different groups like normal, early, and advanced were identified in both datasets. Common DEG among the same groups in both datasets was identified. Further, common DEGs under all three groups were compared to identify common gene signatures.

### Network analysis and module extraction

Identified gene sets were fed to the STRING and the protein–protein interactions (PPI) were visualized among them using Cytoscape v 3.8.2. Gene pairs with a combined score greater than 0.4 were considered for networking. The top modules were extracted using the MCODE (Molecular Complex Detection) plugin of Cytoscape. Parameters taken for the module extraction were degree cutoff = 2, Node score cutoff = 0.2, K-score = 2, and Max. depth = 100.

### Gene ontology analysis

Further, gene ontology of identified DEGs was processed through three online tools namely gProfiler (https://biit.cs.ut.ee/gprofiler/gost), GoNet (https://tools.dice-database.org/GOnet/), and WEB-based GEne SeT AnaLysis Toolkit (https://www.webgestalt.org/). g: Gost in gProfiler performs over-representation analysis or gene set enrichment analysis and generates statistically significant terms. Similarly, GOnet is a web application that performs GO term enrichment analysis and produces interactive visualization of GO analysis results.

### Expression profile and survival analysis

The expression of identified biomarkers was further validated with TCGA and GTEx datasets of Liver hepatocellular carcinoma in 369 tumors and 160 normal cases using the GEPIA (http://gepia.cancer-pku.cn) tool. Log2FC cut-off and *p*-value cut-off were taken as 1 and 0.01 respectively. Tumor samples were indicated in red color while normal samples were indicated in grey color. Survival analysis was also performed using the GEPIA tool. Overall survival with a median group cut-off at a 95% confidence interval and a cut-off high as low as 50% was taken as criteria. Overall survival analysis for each gene selected based on the MCODE score was performed.

### Transcription factor prediction analysis

Transcription factors and micro-RNAs related to biomarkers have been identified using the Network analyst tool (https://www.networkanalyst.ca/). Networkanalyst is used for gene/protein list. The gene list is pasted in the gene list input tab, Homo sapiens were selected and uploaded for analysis.

## Results

### Unveiling 50 core DEGs in gallbladder cancer progression

**Figure 2:**
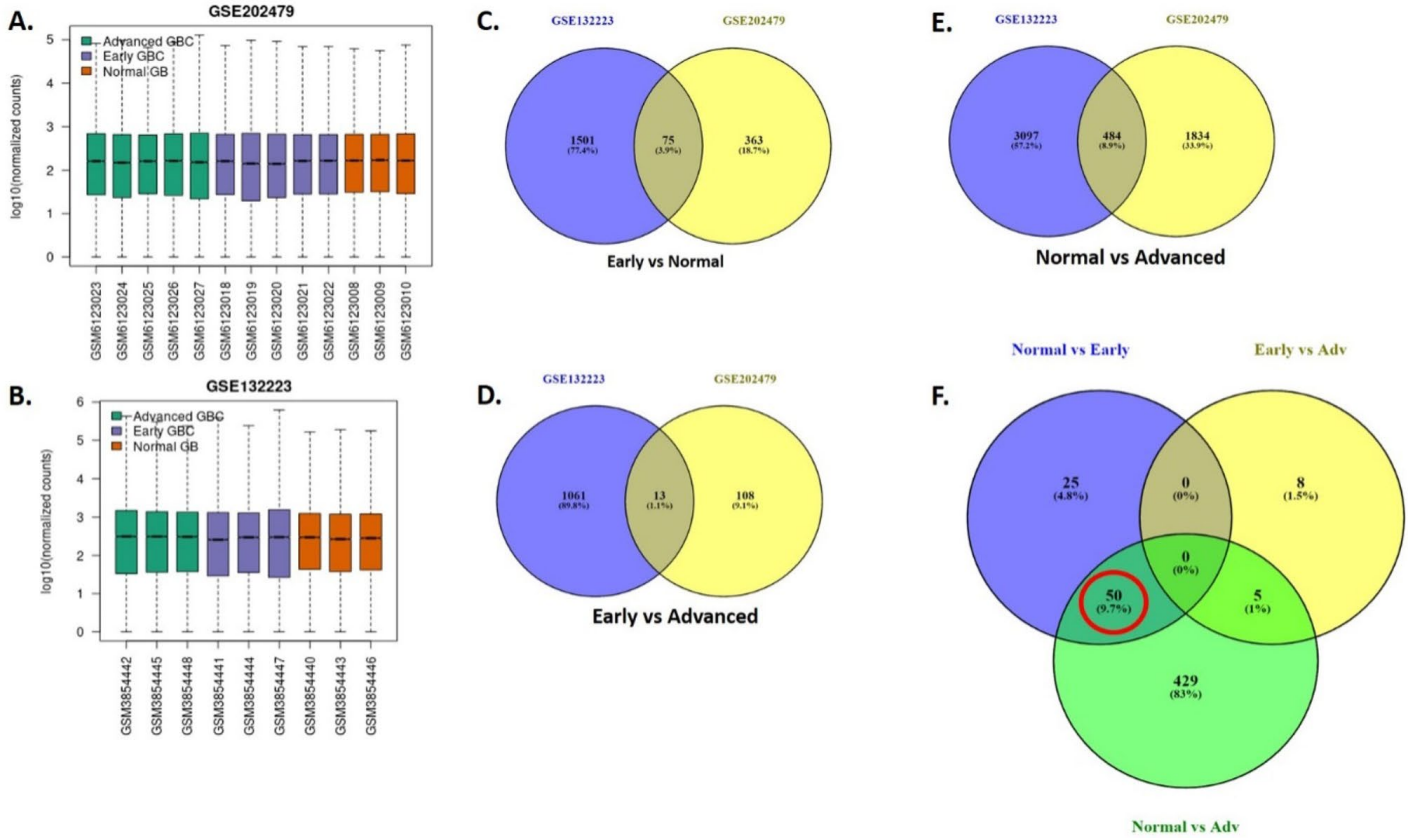
GEO datasets and Common DEGs: (**A**) Box Plot showing the normalization of advanced, Early, and Normal Gallbladder Samples in the GEO datasets GSE202479. (**B**) Box Plot showing the normalization of advanced, Early and Normal Gallbladder samples in the GEO datasets GSE132223. (**C**) Venn diagram showing the common DEG identified for early stage between the both datasets GSE202479 and GSE132223. (**D**) Venn diagram showing the common DEG identified for the early and advanced stage between both datasets GSE202479 and GSE132223. (**E**) Venn diagram showing the common DEG identified for the advanced stage between both datasets GSE202479 and GSE132223. (**F**) Venn diagram showing the common DEG (highlighted in red circle) which are expressed in early as well as advanced stage of GBC.

The expression profiling of both datasets with GSE IDs GSE202479 and GSE132223 were analyzed with GEOquery and limma package in GEO2R. The box plot gives insights into the normalization of data in both datasets (Fig. 2A,B). The median expression of GSE202479 as well as GSE132223 is more positively skewed in the advanced stage of GBC, positively skewed in the early stage of GBC, and almost falls on a median in normal cases. Although most of the data fall centrally, in an advanced stage of gallbladder cancer the data are spread towards maximum. Our analysis identified sets of common DEGs in both the dataset based on both magnitude of change (log2 fold change + /−1) and statistical significance (adjusted *p*-value < 0.05) in three comparisons: Early versus Normal (75 DEGs) (Fig. 2C), Normal versus Advanced (484 DEGs) (Fig. 2D), and Early vs. Advanced (13 DEGs) (Fig. 2E). Further comparison employing Venn diagrams revealed 50 significantly differentially expressed genes (DEGs) which will serve as early biomarkers of GBC as these are present in the early stage as well as advanced stage of GBC (Fig. 2F), (Supplementary file 1).

### Construction of network, analysis and extraction of modules

**Figure 3:**
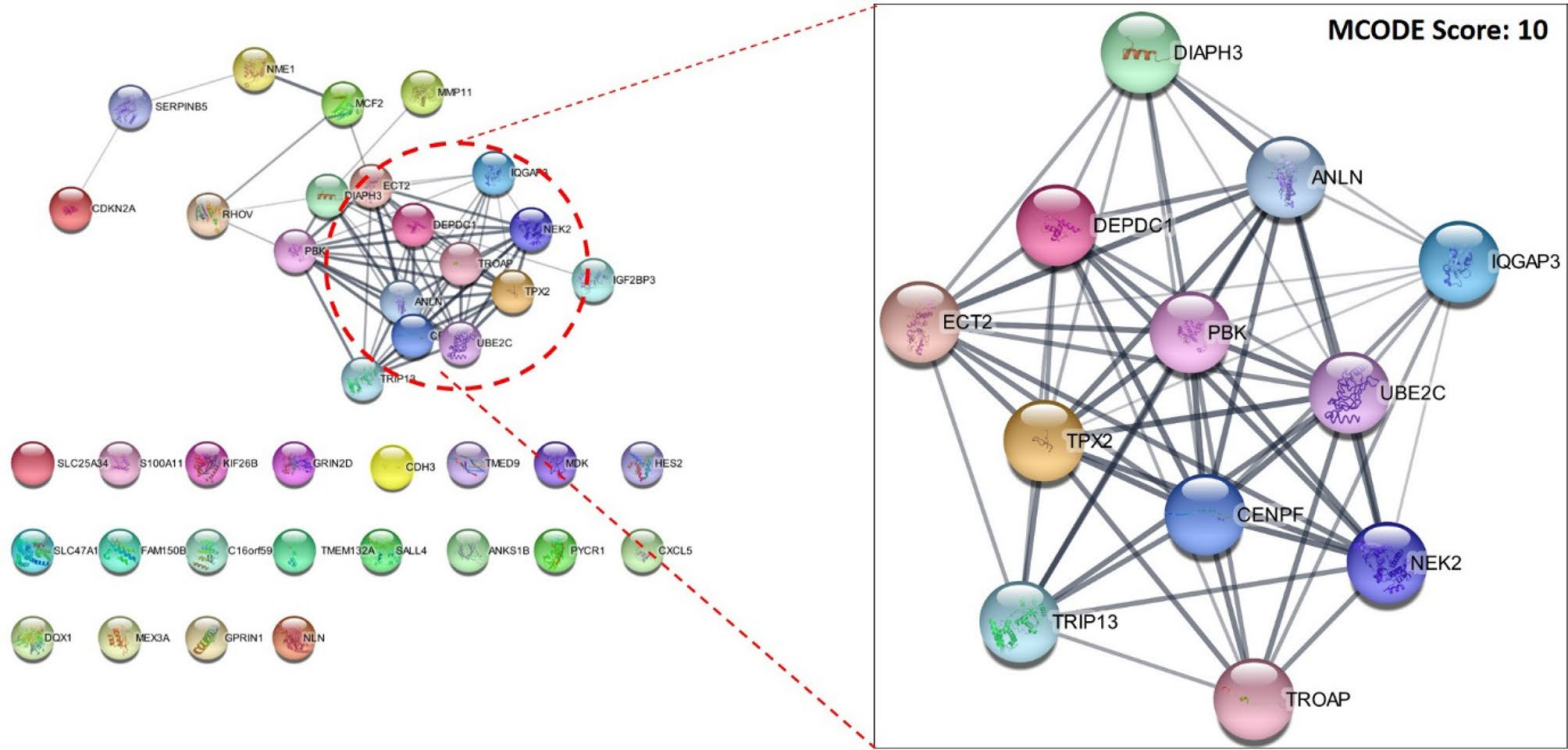
Protein–Protein Interaction network analysis: Network analysis for 50 DEGs common between early and advanced stages of GBC was performed using STRING (Left Side), combined score greater than 0.4 was taken as criteria for network construction. Subsequently, the MCODE plugin of Cytoscape revealed the Sub-network with the highest MCODE Score i.e. 10 (Right Side).

All 50 early-stage biomarkers were then subjected to STRING analysis (https://string-db.org/) and network analysis by Cytoscape. STRING analysis resulted in a protein–protein interaction network with a combined score > 0.4. The further network was visualized in Cytoscape and module extraction was performed using the MCODE plugin. Our data analysis revealed a highly interconnected module consisting of 12 DEGs with an MCODE score of 10, suggesting a potential biomarker for gallbladder cancer progression. These 12 DEGs include DIAPH13 (Diaphanous-related formin (mDia1) protein), DEPDC (Deep domain-containing protein 5), ANLN (Anillin), IQGAP3 (IQ motif-containing protein/GTPase activation protein 3), ECT2 (Epithelial cell transforming 2), PBK (PDZ-binding kinase), UBE2C (Ubiquitin-conjugating enzyme E2C), TPX2 (Transcription factor X2), CENPF (Centromere protein F), NEK2 (NIMA-related kinase 2), TRIP13 (truncated brain-derived neural survival factor), and TROAP (Tax1-related protein-interacting protein 3). Subsequently, the 12 genes within this module with the highest individual MCODE scores were selected for further pathway analysis shown in Fig. 3, (Supplementary file 2).

### Gene ontology (GO)analysis of key DEGs in gallbladder cancer

**Figure 4:**
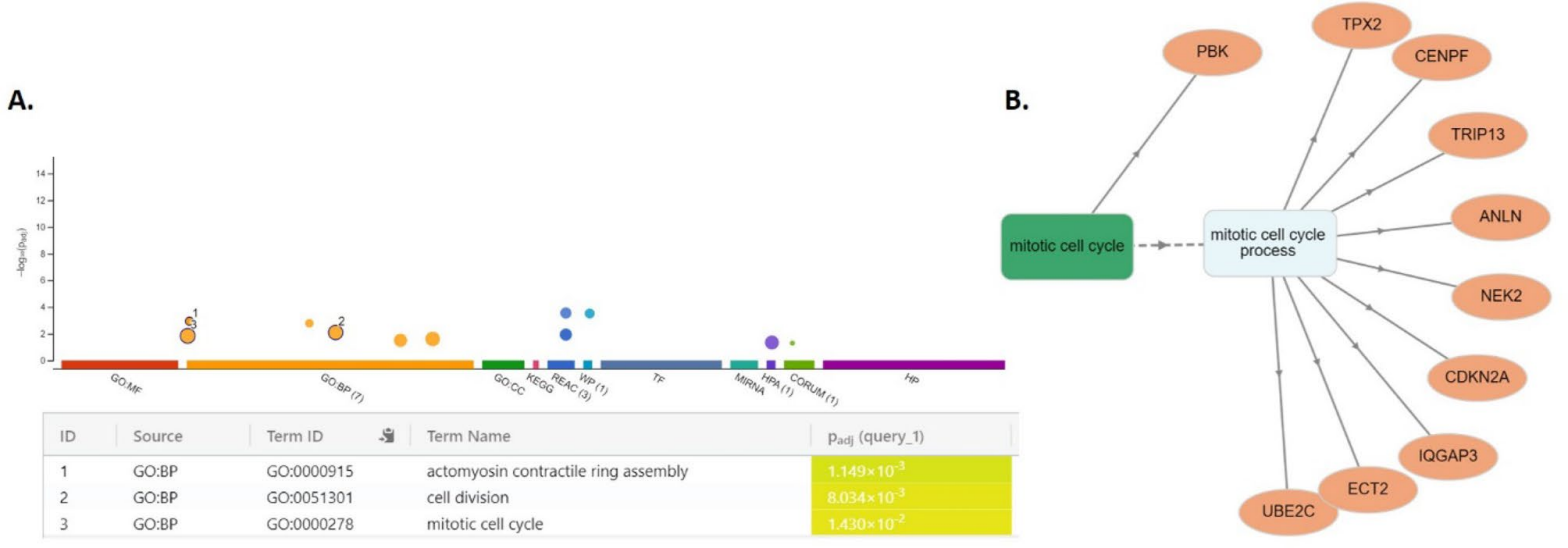
Gene Ontology (GO) analysis: GO analysis for early-stage biomarkers (DEGs common between early and advanced stages of GBC) was performed using gProfiler and GOnet. (**A**) GO analysis using gProfiler (https://biit.cs.ut.ee/gprofiler/gost), shows the significant biological process, molecular function, and cellular component for early-stage biomarkers. (**B**) Similarly GO analysis using GoNet (https://tools.dice-database.org/GOnet/) shows the the network of biological processes and DEGs involved in that. Analysis from both tools shows the mitotic cell cycle as a significant biological process.

Unveiling the functional underpinnings of gallbladder cancer progression, Gene Ontology analysis of these 12 high-scoring STRING modules (MCODE score 10) was performed. GO analysis performed through two different tools revealed the same result. Gene Ontology analysis revealed a remarkable enrichment for processes critical for cancer cell proliferation and cell motility (Fig. 4). These genes, intimately linked to actomyosin contractile ring assembly, cell division, and the mitotic cell cycle, seem to likely play a pivotal role in orchestrating the uncontrolled growth and invasive behavior that is a hallmark of cancerous cells. This convergence of key regulatory functions in a single module highlights its potential as a promising therapeutic target for combating this aggressive disease.

### Validation of identified biomarkers in liver hepatocellular carcinoma using TCGA and GTEx datasets

To validate the potential clinical significance of the identified genes, we further investigated their differential expression in liver hepatocellular carcinoma (HCC) using the GEPIA platform (http://gepia.cancer-pku.cn/) Leveraging the robust datasets of TCGA and GTEx, comprising 369 tumors and 160 normal liver tissue samples, we implemented stringent criteria of absolute log2 fold change (|log2FC|) ≥ 1 and *p*-value < 0.01 to identify differentially expressed genes (DEGs) with high confidence.

**Figure 5:**
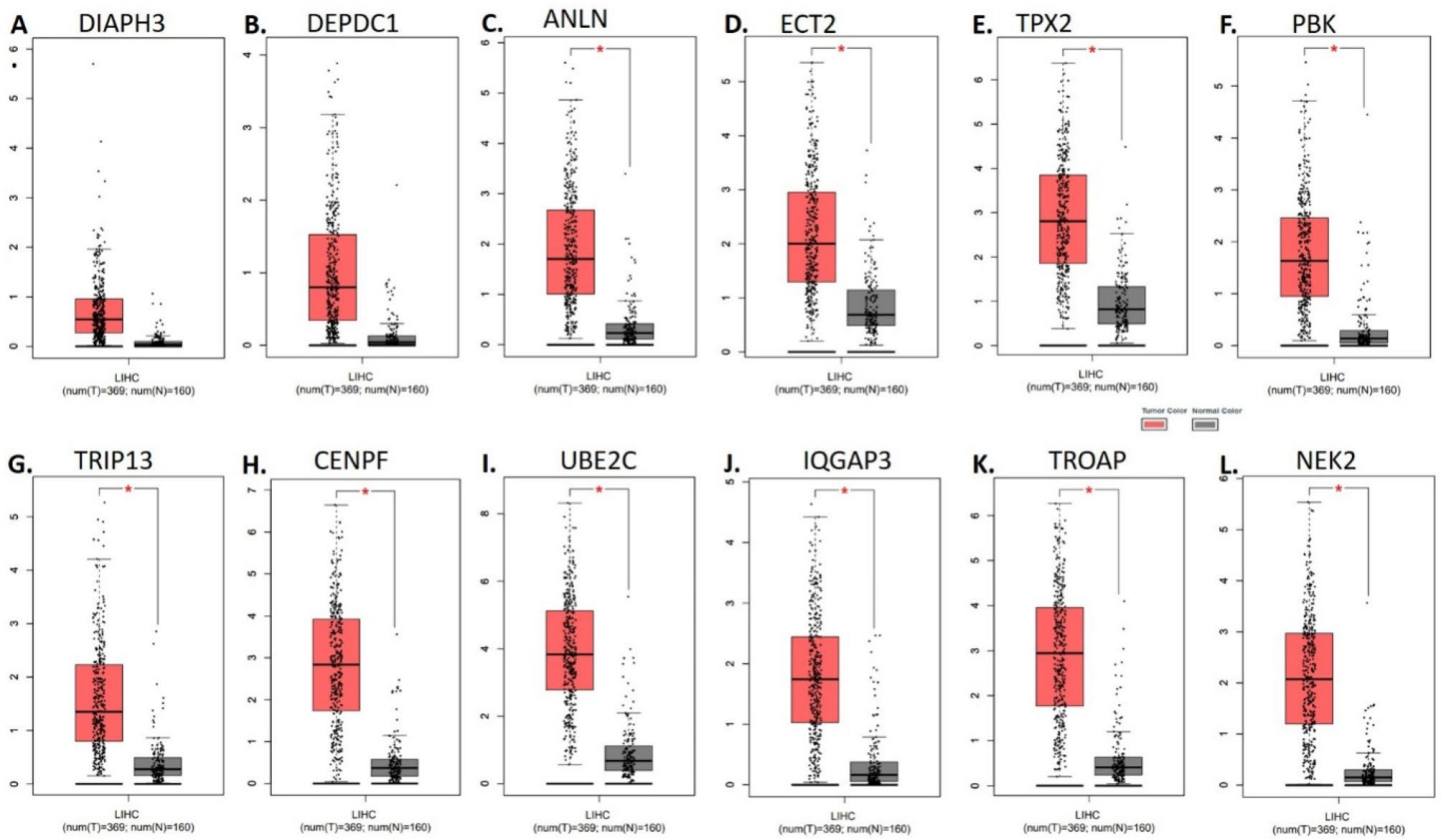
Expression profile of early-stage biomarkers in TCGA and GTEx datasets: The mRNA expression of all 12 early-stage biomarkers in TCGA and GTEx datasets was analyzed in the Liver Hepatocellular Carcinoma (LIHC) datasets using the GEPIA tool. Results showed significant upregulation of almost all 12 DEGs. Note: Red Star (*) denotes significant biomarkers.

This rigorous approach yielded a comprehensive landscape of gene expression alterations associated with HCC progression. Tumor samples, visually distinguished by the red color on the GEPIA platform, exhibited distinct expression patterns compared to their grey-hued normal counterparts. Significant biomarkers are marked with a red star sign in the box plot of expression analysis which are for the genes ANLN, ECT2, TPX2, PBK, TRIP13, CENPF, UBE2C, IQGAP3, TROAP, and NEK2 while insignificant markers are DIAPH3 and DEPDC1 (Fig. 5). This stark contrast highlights the altered transcriptional activity within the cancerous context, providing a valuable starting point for further investigation.

### Biomarker-driven survival analysis

**Figure 6:**
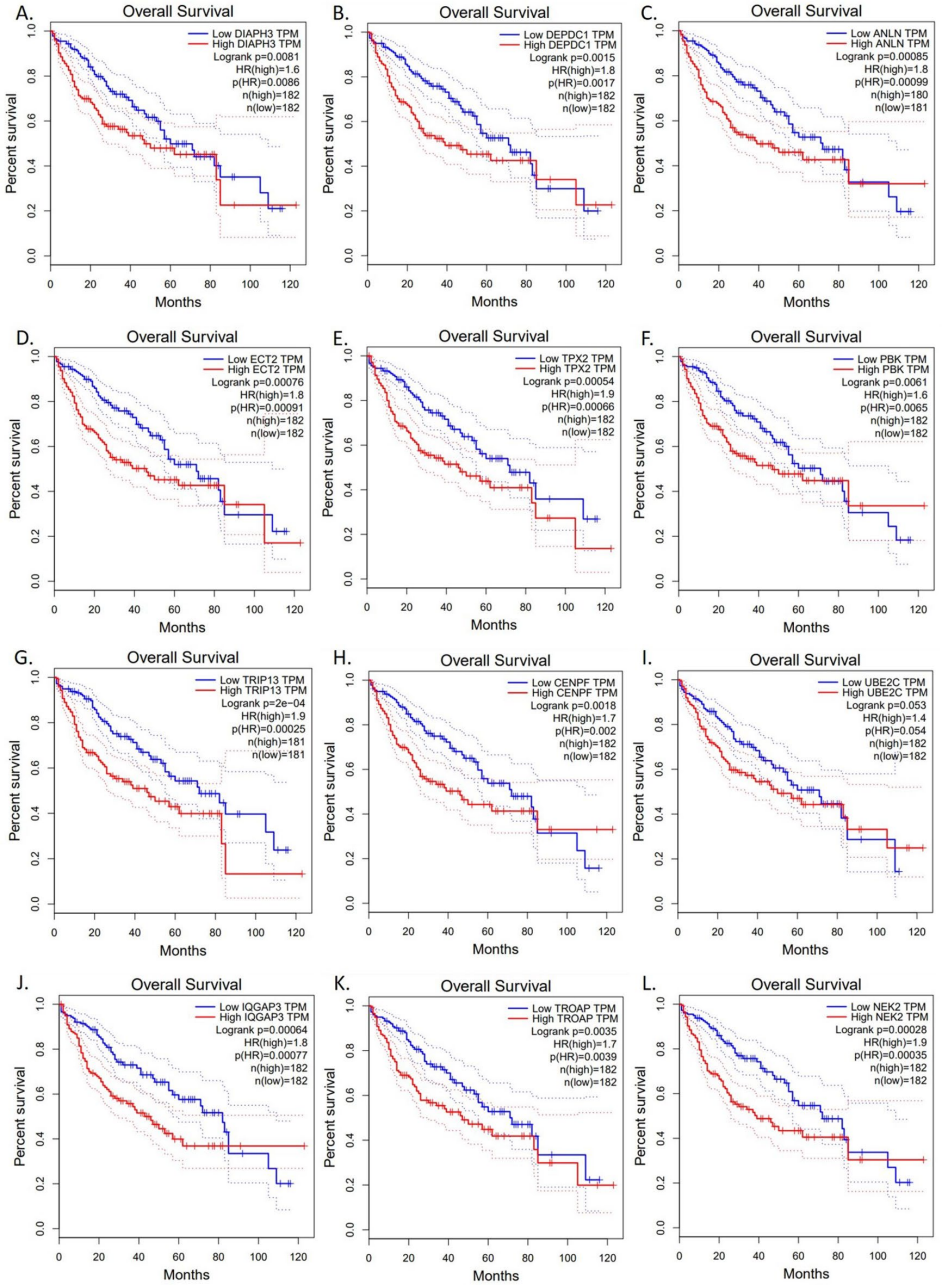
Survival Graph for significant genes: All the early-stage biomarkers were analyzed for the overall survival effect on GBC using the GEPIA tool. The hazard ratio for biomarkers TPX2 and TRIP13 was significantly higher among all DEGs.

Survival analysis of all significant biomarkers reached the point of the hazard ratio of each gene involved in the disease and the graphs of the survival are depicted in Fig. 6. High expression of gene UBE2C is associated with a 1.4-fold increased risk of death, highlighting its potential as a prognostic marker. Each unit increase in genes DIAPH3, and PBK expression is associated with a 1.6-fold higher hazard of death, suggesting its potential role in cancer progression. Patients with tumors exhibiting high levels of genes CENPF, and TROAP have a 1.7 times greater risk of mortality, underscoring its importance in risk stratification. The 1.8-fold increase in hazard ratio associated with high expression of genes DEPDC1, ANLN, ECT2, and IQGAP3 marks a significant leap compared to other identified genes, highlighting the potent influence of these genes on patient survival and its potential as a critical prognostic marker. Most interestingly genes TPX2, TRIP13, and NEK2 demonstrated a striking effect, with its over-expression translating to a 1.9-fold increase in hazard ratio, suggesting its critical role in patient outcomes. Genes having corresponding hazard ratio are identified and listed in Table 1.

**Table 1:** Genes with a corresponding hazard ratio.

Gene	HR	*p*-value
UBE2C	1.4	0.054
DIAPH3	1.6	0.0086
PBK	1.6	0.0065
CENPF	1.7	0.002
TROAP	1.7	0.0039
DEPDC1	1.8	0.0017
ANLN	1.8	0.00099
ECT2	1.8	0.00091
IQGAP3	1.8	0.00077
TPX2	1.9	0.00066
TRIP13	1.9	0.00025

### Network analysis reveals interconnectedness of key biomarkers in gallbladder cancer

**Figure 7:**
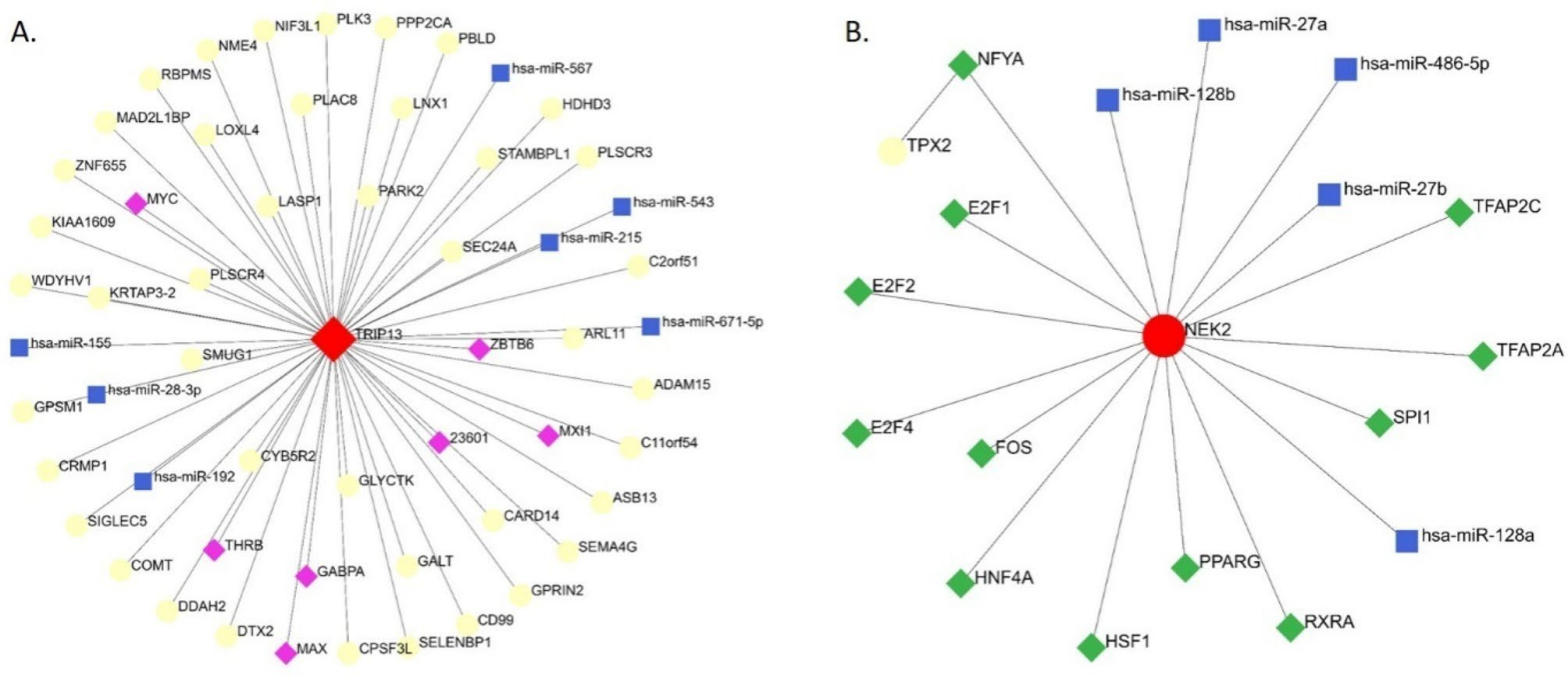
Transcription factor and miRNA prediction: TF and miRNA prediction analysis was performed using the Networkanalyst tool. Analysis revealed two separate networks for TRIP13 (**A**) and NEK2 (**B**). Biomarkers TRIP13 and NEK2 are shown in red, miRNA is shown in blue square while TF is shown in green or purple diamond.

Building upon the identification of potent prognostic biomarkers in liver cancer (TPX2, TRIP13, and NEK2), we delved deeper into their regulatory networks using NetworkAnalyst, a powerful tool for exploring biological interactions depicted in Fig. 7. This comprehensive analysis revealed a complex interplay between transcription factors (TFs), microRNAs (miRNAs), and these key genes, shedding light on the molecular mechanisms underlying their impact on patient survival. TRIP13 emerged as a central hub, with interactions identified for 42 TFs, 7 miRNAs, and 7 additional genes. This extensive network suggests a multifaceted regulatory landscape, where TRIP13's influence on survival likely involves diverse transcriptional and post-transcriptional mechanisms. NEK2 also displayed a notable network, encompassing interactions with 12 TFs and 5 miRNAs. This intricate network highlights the potential for NEK2's survival impact to be modulated by various regulatory factors. TPX2, while having fewer interactions with 1 TF, still offers valuable insights into its potential regulatory context. Understanding this interaction may provide crucial information on TPX2's role in liver cancer progression and survival.

## Discussion

As the most common digestive tract malignancy, GBC is a very aggressive, asymptomatic, and fatal cancer in both women and men worldwide. To date, there is no such early-stage potential diagnostic and prognostic biomarker that has been identified to improve patient outcomes. Due to the absence of early-stage biomarkers, patients are always diagnosed at a very late stage when treatment outcomes are very poor. Further, very few bioinformatics studies have been performed in the field of GBC to identify potential biomarkers associated with GBC progression. Thus, our present in silico analysis study using different bioinformatics tools may help to reveal some early-stage biomarkers associated with GBC. For the study we found two datasets GSE IDs GSE202479 and GSE132223, upon analysis, we identified 50 DEG genes that play a crucial role in the progression of GBC. Further using a network-focused method like STRING and Cytoscape, we examined how these genes are interconnected to each other, finding a central group of 12 key genes with high MCODE Score (10). Most interestingly three genes TRIP13, NEK2, and TPX2 were identified as key players linked to the development and progression of gallbladder cancer. Further, our data is corroborated by a recent study by Yang et al. 2019., and Baolong et al., 2022 explain the significance of TRIP13, NEK2, and TPX2 in Human cancer and prognostic significance in colorectal cancer respectively. Thyroid Transcription Factor 1-Interacting Protein 13 plays a crucial role in cell division and the mitotic spindle checkpoint, ensuring accurate chromosome segregation during cell cycle progression^11^. A previous study indicates that TRIP13 is elevated in Breast Cancer tissues, especially in lung metastatic lesions, and it can facilitate the proliferative and migratory abilities of BC cells^12^. TRIP13 and Mad2 overexpression are linked to multiple cancers. TRIP13 reduces Mad2-induced mitotic delay while lowering TRIP13 worsens Mad2 effects. Decreasing TRIP13 and increasing Mad2 inhibits cell and tumor growth, suggesting TRIP13 inhibition as a potential therapy^13^. NIMA-Related Kinase 2 is critical for centrosome maturation and microtubule dynamics and is essential for cell division and motility^14^. NEK2 manipulation affects cell behavior in gastric cancer, influencing growth and migration in vitro, and tumor growth in vivo, emphasizing its pivotal role in malignancy^15^. TPX2 is a microtubule-associated protein that is required for mitotic spindle assembly and function. TPX2 has a nuclear localization sequence (NLS) and is nuclear-localized during interphase; in mitosis, it localizes to spindle microtubules, with an enrichment toward the spindle poles^16^. Furthermore, microRNA prediction revealed that miR-29c-3p regulates TPX2 to induce cell proliferation in osteosarcoma through the AKT signaling pathway^17^. Non-coding RNA plays an important role in various biological processes related to cancer progression. In this context, predicting and studying the potential human lncRNA-miRNA interactions related to GBC will provide the underlying mechanism of GBC progression. Several computational models like DCAMCP (Deep learning model based on Capsule network and Attention Mechanism for Molecular Carcinogenicity Prediction)^18^, scAAGA (Single Cell data analysis using Asymmetric Autoencoder with Gene Attention)^19^, and MDA-AENMF (Metabolite-Disease Associations based on Auto-Encoder and Non-negative Matrix Factorization)^20^, GCNCRF (Graph Convolutional Neural network and Conditional Random Field)^21^, NDALMA (Network Distance Analysis model for LncRNA-miRNA Association prediction)^22^ can be employed to study the carcinogenicity prediction, single cell clustering and metabolite-disease prediction can also be employed to further enrich the study. Kaplan–Meier survival curve analysis demonstrated that GBC patients with a high expression of TRIP13, NEK2, and TPX2 had a poor prognosis, suggesting that TRIP13, NEK2, and TPX2 may be a potential prognostic factor for GBC patients (Fig. 6). Therefore, TRIP13, NEK2, and TPX2 can be used as a novel prognostic biomarker to identify, distinguish, and predict GBC progression. This study, using a network-centric approach, reveals the complex interaction among differentially expressed genes (DEGs) in gallbladder cancer (GBC), highlighting the crucial roles of TRIP13, NEK2, and TPX2 in steering the disease's development and prognosis. By further understanding this molecular interplay, we can design innovative therapeutic strategies to enhance patient outcomes in the battle against GBC. However, studies in a larger patient cohort and in-depth basic mechanistic studies would provide next-level information and implications of TRIP13, NEK2, and TPX2 genes. Our study would pave a strong pathway for future detailed studies on TRIP13, NEK2, and TPX2 to designate as biomarkers for early diagnosis of GBC.

Further, the present study lacks ODE-based theoretical modeling studies which will help in further understanding of regulatory mechanism. For example, the SWATH-MS technique can be employed to understand the complexity of signaling and the quantitative picture of the dynamic interplay among proteins^23–26^. Despite these models, DMFGAM and GCNAT can also be used to further enrich the study^27,28^. Single Cell Transcriptomics can further resolve the transcriptome heterogenicity among cancerous cells and between cancer and normal cells which will further deepens our molecular understanding of GBC^29,30^.

### Supplementary Information

Supplementary Information 1.

Supplementary Information 2.

## Data Availability

The detailed datasets analyzed during the current study are available with the corresponding author. In the future, it will be made available on reasonable request. Few data were used under license for the current study. Therefore, it is not publicly available. Data are however available from the authors upon reasonable request.
